# Endurance and Speed Capacity of the Korea Republic Football National Team During the World Cup of 2010

**DOI:** 10.2478/v10078-011-0079-9

**Published:** 2011-12-25

**Authors:** Oh Sang Duk, Kim Sung Min, Adam Kawczyński, Paweł Chmura, Dariusz Mroczek, Jan Chmura

**Affiliations:** 1 Department of Athletes Motor Skills, Sport Institute, University School of Physical Education, Wrocław, Poland; 2 College of Physical Education, Hanyang University, Seoul, Korea; 3 Department of Team Sport Games, University School of Physical Education, Wrocław, Poland

**Keywords:** Castrol system, soccer, speed, endurance, game analysis

## Abstract

The aim of the study was to characterize selected indices of endurance and speed of the Korea Republic team with reference to the four best teams during the World Cup of 2010. Five hundred and ninety-nine football players from thirty-two teams participated in the study. All teams played in the 2010 World Cup in South Africa. For the assessment of the players’ motor activity during matches common kinematic test results were recorded using the Castrol Performance Index. The following variables were analysed: total distance covered by the team, distance covered by individual players, maximum running speed and average match running speed for the team and individual players, as well as with division with regard to playing position: defenders, midfielders, strikers. In comparison to the four best teams at the football World Cup of 2010, the Korea Republic players achieved the highest running speed (p≤0.05), and similar levels of covered distance and average match running speed.

## Introduction

Soccer is characterized by high pace and great variation of the game, as well as the use of different energy substrates by players during a game. Short and high intensity activities (sprints, attacks) are combined with work of medium and low intensity (jogging, walking with or without the ball) and even standing. Players’ activity defined by such variables as running speed and covered distance can be monitored by means of new technologies (Barros et al., 2007; Di Salvo et al., 2007; Rienzi et al., 2000). Specificity of match effort has been a topic of many studies and analyses performed in several research centers around the world (Bangsbo, 1994; Buchheit et al., 2010; Chmura et al., 2010; Gregson et al., 2010).

Physical conditioning which includes endurance, strength and speed, constitutes one of the main factors determining success in soccer. It has a direct influence on physical activity of a single player, as well as the whole team. Distance covered by players and teams in a game is one of the objective methods in assessing performance.

Elite soccer players can cover from 10 to 13,5 km during a game, which depends on playing position (Bangsbo et al., 2006; Barros et al., 2007; Di Salvo et al., 2007). The longest distance is covered by midfielders (average 11,5 km), defenders and forwards (average 10,5 km) (Rienzi et al., 2000). It should be underlined that there is a difference with regards to the distance covered: side defenders cover a much longer distance (11 km to 11,5 km), than central defenders which cover between 9 to 10 km (Bangsbo, 1994; Barros et al., 2007).

Modern football is a very dynamic sport discipline. The speed of a player is one of the most important factors influencing the final outcome of the match. Speed of the game is based on two pillars, i.e. motor and cognitive, which are closely related functionally. The effectiveness of the motor pillar is determined by neurophysiological processes, but cognitive speed depends on receiving and processing information by analytical and decision making parts of the cerebral cortex (Hönl, 1996).

The speed of a players movement during a match depends not only on running efficiency and other motor abilities (motor pillar), but also on psychomotor skills such as speed and accuracy of perception, prediction, decision making, choice and speed of response (cognitive pillar).

Players speed activity can be measured by distance covered in sprint, amount of sprints, sprint frequency, as well as maximal and average running speed. Those values depend on playing position. Gregson et al. (2010) reported that English Premier League players cover in sprint from 145 meters (central defender) to 307 meters (wide midfielder) in a single game. The amount of sprints varies from 20 to 41.

### Aim of the study

The aim of the study was to analyze selected indices of endurance and speed of the Korea Republic team with reference to the four best teams during the World Cup of 2010.

### Subjects

Subjects consisted of 599 players from 32 teams which played in the 2010 World Cup in South Africa, with particular consideration to the Korean Republic’s team members, as well as the four best teams, i.e. Spain, Netherlands, Germany and Uruguay.

## Methods

For the assessment of the players’ motor activity during matches, common kinematic test results recorded by means of the Castrol Performance Index were used. With a set of cameras, each movement of every player over the whole field was recorded, and this material was then processed through a special program into quantitative data. Based on this data, the analysis was made of total distance covered by the team, as well as by individual players, maximum running speed and average match running speed for the team and individual players, with regard to the division into formations: defenders, midfielders and strikers, was performed.

Maximal running speed was assessed for each player in each game. Based on the Castrol System, running speed greater than 22 km/h (6,11 m/s) was considered as very high. Maximal running speed, for teams and formation, was calculated as an average from individual highest speeds reached in matches played. Average match running speed was calculated by dividing the distance covered during matches played by total playing time.

### Statistical analysis

The results obtained were subjected to a statistical analysis, where arithmetical average and standard deviations were taken into consideration. Covered distance, maximal running speed and average match running speed were examined using a repeated-measures analysis of variance (RMANOVA). Afterwards, significant results were analyzed by means of simple contrast. The level of significance was accepted as p≤0.05.

## Results

The total distance covered by Korea Republic football players in four matches played at the World Cup of 2010 was 427.80 km – including the goalkeeper. The Koreans covered the longest distance in their win over Greece (2:0) – 108.82 km and the shortest in their heavy defeat to Argentina (1:4) – 103.10 km. The difference between these values, 5,72 km, was statistically significant (p≤0.05). During the last group match, a 2:2 draw with Nigeria (103.10 km), and also in the 1:2 loss to Uruguay in the 116 final (108.36 km), the Korea Republic players covered a significantly longer distance (p<0.05) by 4.43 and 5.26 km respectively, in comparison to the shortest distance covered in the match against Argentina. It should be noted that the total distance covered in the win over Greece and the loss to Uruguay were at the same level.

Table 2 presents the average distance covered by Korea Republic players, excluding the goalkeeper, in comparison to the four best teams of the 2010 World Cup. The longest distance was covered by current world champions Spain, followed by Uruguay and the Netherlands. However, these results were obtained after overtime. Despite this fact, the differences between average values were statistically insignificant. Only Germany’s result was comparable to the one of Korea, as it was obtained without overtime. The distance covered by the Korean players was very close to that of the third place team at the World Cup of 2010 - Germany (Table 2).

From the analysis of particular formations, it appears that during the four matches played, the longest distance was covered by midfielders, followed by forwards and then defenders (Table 3). The midfielders covered the greatest distance in the match against Nigeria - 11.19 ± 0.33 km, and the shortest against Argentina - 10.46 ± 0.29 km. The defenders covered the greatest distance against Uruguay - 9.85 ± 1.15 km, and the shortest, similarly to the midfielders, against Argentina - 9.28 ±0.72 km. Among forwards, the longest distance covered – 9.72 km, was recorded in the match against Nigeria.

Korean players achieved the highest running speed in the game against Greece, and lowest running speed in the game against Argentina (Table 1). The difference between these values was statistically significant (p≤0,05), at the level of 0.81 m/s. The difference between maximal running speed in the defeat to Uruguay and victory over Greece was also statistically significant (p≤0,05) (Tab. 1).

During the World Cup of 2010 Korean players achieved higher maximal running speeds when compared to the top four teams (Table 2). In individual sprints, they were significantly (p≤0.05) faster by 0.17 m/s than current world champions, Spain. The maximal running speeds of the Dutch, German and Uruguayan teams were at a similar level to the Korea Republic players (Table 2).

Maximal running speed achieved by individual formations in matches played is presented in Table 3. The average values of variables examined between the formations showed no significant differences. The highest running speed was recorded by the defenders (6.62 ± 0.58 m/s) and midfielders (7.28 ± 0.18 m/s) in the match against Greece. A significant reduction in the analyzed motor ability was recorded in midfield and forward players in the match against Argentina. The highest running speed (8,34 m/s) was achieved by a midfielder and (8,03 m/s) by a forward in the match against Nigeria.

The highest average match running speed was observed in the 116 final match against Uruguay, and the lowest in the group stage match against Argentina (Table 1). Average values did not show significant differences. Furthermore, the average value of the examined indicator did not differ significantly between Korea Republic and the three first place teams (Table 2). However, in comparison to the fourth team, Uruguay, these values were at the same level (Table 2). Research has shown that midfielders achieved significantly (p≤0.05) higher average match running speed than defenders (Table 3). Among players who played a minimum of 90 minutes at the 2010 World Cup, the highest individual average speed – 2.11 m/s was observed twice in midfielders in the match against Greece and Nigeria.

## Discussion

Based on the analysis of endurance and speed, it is possible to asses the level of physical conditioning. Regarding to covered distance, maximum and average match running speed, Korean Republic team was well prepared in respect to endurance and speed for the 2010 World Cup in South Africa. From among 32 teams at the World Cup (excluding teams which played matches with overtime), Korea Republic achieved the eighth highest result in terms of distance covered by the whole team (106.95 km). The result was better than the Brazilian team (by 6.95 km) and the French team (by 6.23 km). The sport results achieved by Brazilian and French soccer players proved that even teams with excellent football skills without proper endurance are not able to fully use them in prestigious tournaments. In modern soccer it is difficult to win a match using only skills and vice versa. The appropriate balance between technical-tactical skills and physical conditioning must be maintained.

Noteworthy is the fact that regardless of the result in each of the four matches played, Korea Republic team covered a longer distance than their opponents: in the group stage with Greece (by 3.22 km), with Argentina (by 5.94 km), with Nigeria (by 8.23 km) and in 1/16 finals game with Uruguay (by 1.79 km). Covering a longer distance by 8.23 km in a match with Nigeria practically means that each Korean player (excluding goalkeeper) covered on the average a distance longer by 823 m than each Nigerian player. These data indicate an excellent endurance of Korean players, especially in comparison to their opponents.

From analysis of particular formations it appears that the longest distance was covered by midfielders, then forwards and defenders what is confirmed in studies by other authors (Barros et al., 2007, Rienzi et al., 2000). Volume of work performed during a match by the best defenders and forwards is at a similar level and it becomes closer to the distance covered by midfielders. It is the result of applying the “total football” principle where a forward performs the functions of a defender and vice versa.

The World Cup in South Africa proves that players with the best endurance cover in one match a distance from 10.5 to 12.9 km, including defenders from 10.8 to 12.0 km, midfielders from 12.3 to 12.9 km, and forwards from 10.5 to even 11.9 km. Whereas with reference to studies of Rienzi et al. (2000), Barros et al. (2007), Di Salvo et al. (2007) values of covered distance were higher, especially for midfielders.

Bangsbo et al.’s (1994) studies show that there is a high correlation between the covered distance and VO_2max_ (l/min) values simultaneously confirming the significance of aerobic metabolism during a game. A high level of aerobic capacity allows the player to not only cover a longer distance but also to develop higher intensity during a match and maintain it for a longer period of the game, more frequent performance of sprints, and a greater acceleration. A player with better aerobic capacity has a better tolerance and resistance to increasing fatigue and recovers faster during and after the game (Bangsbo et al., 2006; Mohr et al., 2010). Moreover a player reaches the psychomotor fatigue threshold at higher effort intensities which allows to play longer in the psychomotor comfort zone (Chmura et al., 2010). It must be underlined that the distance covered during a game also depends on: tactical assumptions, formation, motivation, opponent and game result.

In the match against Argentina endurance and speed of the Korean players decreased. Decreased game dynamics and lower game activity in all formations might be influenced by changing of climate zone, changes in temperature and altitude above sea level. The first game Korea Republic played against Greece in a wet, subtropical climate of Port Elizabeth located at seaside, few meters above sea level at a temperature of 19 – 20°C. A recent study reported that hyperthermia and dehydration are also important factors causing a decrease in sprint ability and pronounced reduction in high-intensity running during a football match (Mohr et al., 2010).

Five days later a match against Argentina was played in a dry, subtropical zone in the mountains of Johannesburg, 1753 m above sea level, at a temperature of 10°C and decreased atmospheric pressure. At this height the partial pressure of oxygen in the blood is lower and it causes a decrease of volume of oxygen delivered to the tissues and working muscles. There is much scientific evidence indicating a decrease of work ability under condition of lower atmospheric pressure (Wehrlin et al., 2006). A decreasing effect of low temperature on muscle speed and maximal power output is also reported (Drinkwater, 2008).

It may be assumed that a several day long stay in the mountains before the match with Argentina was insufficient for full acclimatisation of the body to the mountain climate. Wehrlin et al. (2006) examinations showed a reduction in endurance of 14.5 % for every 1000 metres above sea level. In our research there was a reduction in endurance in Korean players of 5.26%. Despite this fact, the Korean players covered a greater distance than the Argentineans by 5.94 km. This indicates that the Korean players had better endurance than the Argentinean team prior to competition. It is probable that the reasons for the defeat to Argentina can be found in weaker tactical and technical skills of the Korean team. This may be reflected, for example, in the number of passes and their efficiency. In the analysed match the Argentineans performed 35.6 % more passes than Koreans and the efficiency was higher by 12 %. The clearest evidence for the difference in football skills between the teams was the short passes efficiency, which was 20 % higher for Argentineans. It must be underlined that better physical conditioning gives players a psychomotor comfort and allows to use all technical and tactical skills (Chmura et al., 2010).

One of the basic indicators of team game dynamics is maximum running speed. Korean players, in the four matches played, achieved the highest maximal running speed (7.26 m/s) when compared to the best teams of the World Cup of 2010. This is a much higher value than the high speed norm set in the Castrol System. The Koreans were faster on average by 0.17 m/s than current world champions Spain (7.09 m/s). In practice, this means that after achieving maximum speed, they would win one second sprint races by an average of 17 cm. This fact confirms a very high level of speed of Korean players during the World Cup of 2010. In current literature, maximum running speed and distance covered at high speed are the main factors determining physical conditioning of soccer teams (Gregson et al., 2010).

However, the very good speed preparation of the Korea Republic team was not fully reflected in the final result. The Spanish players made up for their lack in maximum speed in comparison to other teams with football skills, which can be reflected, among others, in the highest number of passes completed during the tournament (4752) and highest passing efficiency – 80% (3803 successful passes). Their games were dominated by quick, short passes, mostly first ball, high passing frequency and ball possession.

The Korean team achieved its highest maximal running speed in the first group match with Greece (6.98 m/s). During this match, six players (three defenders and three midfielders) achieved their record speeds, which were not repeated in later matches. This data indicates optimum team dedication to achieving the best possible result in this match. Korean midfielders (7.10 m/s) were faster then defenders (6.93 m/s) by 0.17 m/s. Speed difference between those formation gets smaller during the World Cup of 2010. This is confirmed by the fact that the maximum running speed recorded by the six fastest defenders (from 8.06 to 8.77 m/s) and midfielders (from 8.15 to 8.75 m/s) were at a comparable level. This means that even faster athletes are playing in defence, especially side defenders.

Overall sporting success in football is not decided solely by maximum speed achieved in sprint races, but also by average running speed achieved by players during the match. This depends on distance covered and playing time. Comparable values for average match speed between Korea, Spain, the Netherlands and Germany may indicate optimal physical conditioning, strong determination and commitment.

## Conclusions

1. In comparison to the four best teams at the football World Cup of 2010, the Korea Republic players achieved the highest running speed, and similar levels of endurance and average match speed, which indicates optimal physical conditioning for the competition.
2. Running speed and distance covered during top level matches should provide the basis for planning the training process of elite soccer teams.

## Figures and Tables

**Table 1:** Maximal running speed and average match running speed in particular games of Korean players during the World Cup of 2010.

No.	Game	Score	Maximal running speed [m/s]	Average match running speed [m/s]
1.	Korea Republic - Greece	2:0	6.98 ± 0.48	1.92 ± 0.15
2.	Argentina – Korea Republic	4:1	6.17 ± 0.57	1.83 ± 0.13
3.	Nigeria - Korea Republic	2:2	6.51 ± 0.96	1.90 ± 0.18
4.	Uruguay - Korea Republic	2:1	6.42 ± 0.46	1.93 ± 0.18

* - goalkeeper included

**Table 2:** Covered distance, maximal running speed and average match running speed of Korean players in comparison to the best four teams of World Cup of 2010 (goalkeeper excluded).

No.	Team	Distance covered by player [km]	Maximal running speed [m/s]	Average match running speed [m/s]
1.	Spain	10.62 ± 1.19	7.09 ± 0.70	1.91 ± 0.20
2.	Netherlands	10.33 ± 1.12	7.27 ± 0.57	1.84 ± 0.16
3.	Germany	10.33 ± 0.98 **	7.23 ± 0.73	1.94 ± 0.17
4.	Uruguay	10.56 ±1.16	7.24 ± 0.57	1.90 ± 0.20
5.	Korea Republic	10.28 ± 0.90 **	7.26 ± 0.53	1.90 ± 0.14

* - only players which played 90 min at least.

** - distance covered - without overtime

**Table 3:** Covered distance, maximal running speed, average match running speed of Korean defenders, midfielders and forwards during the games of Word Cup of 2010.

No.	Formation	Distance covered by player [km]	Maximal running speed [m/s]	Average match running speed [m/s]
1.	Defenders	9.57 ± 0.80	6.93 ± 0.32	1.80 ± 0.13
2.	Midfielders	10.91 ± 0.40	7.10 ±0.75	2.08 ±0.14
3.	Forwards	9.69 ± 0.62	7.15 ± 1.19	1.90 ± 0.20
